# Efficacy and safety of radiofrequency ablation for secondary hyperparathyroidism: a systematic review and meta-analysis

**DOI:** 10.2478/abm-2024-0036

**Published:** 2024-12-16

**Authors:** Mengyuan Li, Hongwei Jiang, Yunchang Wang, Fujun Li

**Affiliations:** Department of Ultrasound, The First Affiliated Hospital, and College of Clinical Medicine of Henan University of Science and Technology, Luoyang, Henan Province, 471003, China; Department of Endocrinology, The First Affiliated Hospital, and College of Clinical Medicine of Henan University of Science and Technology, Luoyang, Henan Province, 471003, China; Department of Stomatological Center, The First Affiliated Hospital, and College of Clinical Medicine of Henan University of Science and Technology, Luoyang, Henan Province, 471003, China

**Keywords:** chronic kidney disease, meta-analysis, radiofrequency ablation, secondary hyperparathyroidism, systematic review

## Abstract

**Background:**

Secondary hyperparathyroidism (SHPT) is a common complication of chronic kidney disease (CKD) that affects approximately 90% of end-stage renal disease and poses a significant threat to long-term survival and quality of life in patients.

**Objectives:**

To assess whether radiofrequency ablation (RFA) is a productive and low-risk treatment for hyperparathyroidism secondary to CKD.

**Methods:**

Embase, Web of Science, Cochrane Library, and PubMed were searched independently by two authors. The results after RFA and baseline biochemical indicators were compared, and parathyroid hormone (PTH), serum calcium, and serum phosphorus levels were the major outcomes.

**Results:**

Four retrospective studies were screened out from 147 original literature and involved 118 cases. After RFA, serum PTH levels (1 d standardized mean difference [SMD] = −2.30, 95% confidence interval [CI] = from −3.04 to −1.56, *P* < 0.0001; 6 months SMD = −2.15, 95% CI = from −3.04 to −1.26, *P* < 0.0001; 12 months SMD = −2.35, 95% CI = from −3.52 to −1.17, *P* < 0.0001), serum calcium levels (1 d SMD = −1.49, 95% CI = from −2.18 to −0.81, *P* = 0.0001; 6 months SMD = −1.09, 95% CI = from −1.51 to −0.68, *P* < 0.0001), and serum phosphorus levels (1 d SMD = −1.37, 95% CI = from −1.67 to −1.07, *P* < 0.0001; 6 months SMD = −1.06, 95% CI = from −1.35 to −0.78, *P* < 0.0001) decreased significantly.

**Conclusions:**

RFA, the newest thermal ablation technique, can effectively and safely treat hyperparathyroidism secondary to CKD. Hoarseness is the most common complication but is reversed within 6 months.

Secondary hyperparathyroidism (SHPT) is a common complication of chronic kidney disease (CKD) that affects approximately 50% of the patients with stage 3 or 4 CKD and approximately 90% of those with end-stage renal disease [1,2,3]. As a result of hemodialysis, the prevalence of SHPT exceeds 32% [4]. Persistently increased parathyroid hormone (PTH) levels and parathyroid hyperplasia are the characteristics of SHPT and are mainly caused by hypocalcemia, hyperphosphatemia, and vitamin D deficiency [5,6,7]. Furthermore, the excessive secretion of PTH leads to disorders of calcium and phosphorus metabolism and an increased risk of cardiovascular-related mortality [4, 8]. Clinical features include bone and joint lesions, vascular and soft tissue calcification, urinary lesions, and peripheral neurological and psychiatric lesions, ultimately affecting both long-term survival and the quality of the patients' life severely [9, 10].

Pharmacological treatment exerts a desirable therapeutic effect in the early stages of SHPT but with significant limitations. For instance, drug-refractory SHPT occurs in some patients when the enlarged parathyroid gland is >500 mm^3^ in volume or >1 cm in length, especially after 5–10 years of hemodialysis, and approximately 15% of the patients with drug-refractory SHPT require parathyroidectomy (PTX) [11]. However, the high risk associated with surgery and anesthesia and postoperative complications result in increased difficulty in adopting conventional surgical approaches [12].

Thermal ablation, an advanced minimally invasive treatment technique, has recently become a promising treatment option. It is the combination of several options such as microwave ablation, radiofrequency ablation (RFA), high-intensity focused ultrasound, and laser ablation [13,14,15,16,17]. The advantages of thermal ablation are the ease of operation, faster recovery, higher tolerance by patients, shorter hospitalization, fewer complications, and a shorter recovery period than surgical treatment [18, 19]. Thermal ablation offers a new strategy for the nonsurgical treatment of various diseases, such as liver cancer, renal cancer, and thyroid nodules, with the outcomes and expectations being the same as those of conventional surgery [13, 20, 21].

Although research has shown that thermal ablation can be used to treat refractory SHPT [12, 22, 23], a comprehensive review of RFA in treating SHPT has not been reported so far. Therefore, this systematic review and meta-analysis attempted to assess the efficacy and safety of RFA for treating hyperparathyroidism secondary to CKD based on the published literature.

## Methods

The author's institution does not require ethical approval for this study. This meta-analysis followed the Preferred Reporting Items for Systematic Reviews and Meta-Analyses (PRISMA) statement [24] and Assessing the Methodological Quality of Systematic Reviews (AMSTAR) guidelines. The registration ID is CRD42022361810 (International Prospective Register of Systematic Reviews). Each patient included in the original study signed an informed consent form.

### Search strategy

Two authors (M.L. and F.L.) searched the databases of PubMed, Embase, Cochrane Library, and Web of Science without start time limitation until September 19, 2022. The relevant published literature was searched using the following terms: (Hyperparathyroidism, Secondary OR Secondary Hyperparathyroidism OR Hyperparathyroidisms, Secondary OR Secondary Hyperparathyroidisms) AND (Radiofrequency Ablation OR Ablation, Radiofrequency OR Radio Frequency Ablation OR Ablation, Radio Frequency OR Radio-Frequency Ablation OR Ablation, Radio-Frequency).

### Inclusion and exclusion criteria

The inclusion criteria were as follows: (1) the subjects of the study were *Homo sapiens*. (2) The studies were randomized controlled trials (RCTs) or retrospective studies. (3) The studies involved patients with severe SHPT, defined as high PTH levels (>800 pg/mL), serum calcium levels lower than the normal range, and serum phosphorus levels higher than the normal range. (4) Parathyroid gland hyperplasia was diagnosed via medical imaging. (5) The patients underwent ablation of parathyroid hyperplasia with ultrasound-guided radiofrequency, and the study recorded biochemical index results, such as serum PTH levels, serum calcium levels, and serum phosphorus levels, before and after RFA. (6) The research proved RFA to be effective for treating SHPT. (7) The studies had a minimum 6-month follow-up record after ablation.

The exclusion criteria were as follows: (1) abstracts, meetings, case reports, reviews, and animal studies. (2) Studies on primary or tertiary hyperparathyroidism. (3) Studies without complete data.

### Data extraction and quality assessment

Two investigators (M.L. and F.L.) extracted the data from eligible full-text articles using standard data extraction forms independently, and the extractions were confirmed by a third author (Y.W., with 40 years of experience in ultrasonography). Inconsistencies were resolved via discussion, and a consensus was reached. Serum PTH, calcium, and phosphorus levels were the main outcome indicators in this study. The baseline characteristics of this research were the first author, year of publication, location, study design, sample size, mean age, sex, conducted period, follow-up time point, and major and minor complications. The quality of the RCTs was determined using the Cochrane assessment tool, and the quality of nonrandomized studies was assessed using the Newcastle–Ottawa scale (NOS). Using the Grading of Recommendations Assessment, Development, and Evaluation (GRADE) system to rate the quality of evidence for each outcome [25,26,27], the evidence quality level was downgraded based on study limitations, imprecision, inconsistencies, indirectness, and publication bias; comparisons were rated as high-quality evidence (four plus: ++++).

### Statistical analysis

The meta-analysis used RFA as the segmentation to compare pre-ablation with post-ablation in each follow-up time point with regard to the changes in different biochemical indexes. Pre-ablation served as the baseline, and all subjects served as their controls at varying points in time. The outcomes of continuous variables (serum PTH levels, serum calcium levels, and serum phosphorus levels) at each follow-up time point were assessed and recorded as absolute values. The meta-analysis analyzed the alterations in standardized mean difference (SMD)/mean difference (MD) compared with the baseline and their 95% confidence interval (CI). Cohen's *d* was used to quantify the magnitude of SMD/MD as per Cohen's criteria. The effect sizes were defined as “small, 0.2 ≤ *d* < 0.5,” “medium, 0.5 ≤ *d* < 0.8,” “large, *d* ≥ 0.8,” [28] “very large, *d* ≥ 1.2,” and “huge, *d* ≥ 2.0” [29]. The *x*^2^ test was used to explore the heterogeneity and quality with the inconistency factor (*I*^2^), and *P* < 0.10 or *I*^2^ > 50% indicated significant heterogeneity [30]. In case of no or low heterogeneity, the fixed-effects model (*I*^2^ < 50%) was chosen, and in case of moderate or high heterogeneity, the random-effects model (*I*^2^ > 50%) was applied [31, 32]. Sensitivity analysis is the sequential omission of a study to test the robustness of the main results [30, 33]. Potential publication bias was assessed and quantified using funnel's plot and Egger's test [34, 35]. Statistical differences were expressed at *P* < 0.05, and the nearly symmetric funnel's plot signified the absence of potential publication bias. This meta-analysis was performed using Review Manager, version 5.4 (Nordic Cochrane Center, Oxford, England) and SPSS Statistics Premium 28.0.0.0 (SPSS for Mac OS 28.0, SPSS, Inc. Chicago, IL, USA).

## Results

### Literature search

**Figure 1.**
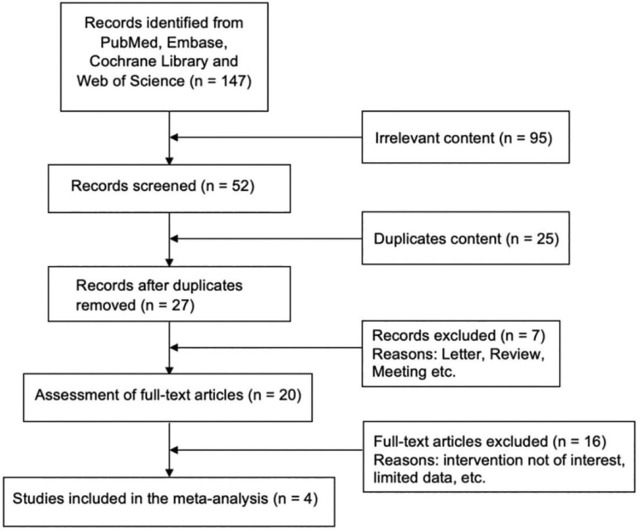
Flow diagram of the literature selection process.

The flow diagram for the literature selection process is shown in **Figure 1**. The initial database search yielded a total of 147 studies. After screening the titles and abstracts, 95 studies were excluded owing to irrelevant content, 25 were excluded because of duplication, and 7 were excluded because of document types. The remaining 20 potentially eligible studies were included in the full-text review. According to the inclusion and exclusion criteria, 4 retrospective studies were included in this meta-analysis [36,37,38,39]. A total of 16 trials were excluded for several reasons (intervention was not of interest or did not present the available data, etc.). Only patients' data in the RFA group were extracted in these 4 studies. In the study by Chen et al. [36], data were extracted only from patients in the SHPT group who underwent RFA without PTX.

The 4 included studies were retrospective studies and comprised 118 patients. The follow-up time points spanned 1 d–26 months. The baseline information of these studies is listed in **Table 1**, and NOS assessments are listed in **Table 2**.

**Table 1.** Basic information of the included studies

**Reference**	**Country**	**Institution**	**Design**	**Sample size**	**Gender (male/female)**	**Mean age (years)**	**Follow-up period (months)**	**Conducted period**	**Follow-up time point (d/months)**	**Complications (*N*)**
Chen et al., [36]	Taiwan, China	The Kaohsiung Chang Gung Memorial Hospital Medical Center in Taiwan	Retrospective study	9	6/3	51.80 ± 16.00	12	2019.04–2020.12	1 month 3 months 6 months 12 months	Hoarseness (2) Neck pain (4)
Ren et al., [37]	China	Zhejiang Provincial People's Hospital	Retrospective study	47	29/18	51 ± 12	28.6 (21.3–36.6)	2014.06–2020.12	1 month 3 months 6 months 12 months 24 months	Hoarseness (6) Hematoma (1) Fever/infection (3) Hypocalcemia (26) Severe hypocalcemia (6)
Zhang et al., [38]	China	Fujian Provincial Hospital	Retrospective study	30	16/14	45.8 ± 13.3	6	2018.01–2021.02	1 d, 7 d 1 month 3 months 6 months	Recurrence (7) Recurrent laryngeal nerve injury (8) Severe hypocalcemia (6)
Qin et al., [39]	China	Nanchong Central Hospital	Retrospective study	32	13/19	53.53 ± 13.64	12	2018.11–2019.05	1 d, 2 d 6 months 12 months	Hoarseness (9) Neck pain (1) Artery injury (1)

**Table 2.** Quality assessment of cohort studies

**Reference**	**Is the case definition adequate?**	**Representativeness of the cases**	**Selection of controls**	**Definition of controls**	**Comparability of cases and controls on the basis of the design or analysis**	**Ascertainment of exposure**	**Same method of ascertainment for cases and controls**	**Nonresponse rate**	**Total scores**
Chen et al., [36]	★	★	★	★	★	★	★	★	8
Ren et al., [37]	★	★	★	★	★	★	★	☆	7
Zhang et al., [38]	★	★	★	★	★	★	★	☆	7
Qin et al., [39]	★	★	★	★	★	★	★	★	8

## Meta-analysis results

### Biochemical indexes and complications

#### PTH level

**Figure 2.**
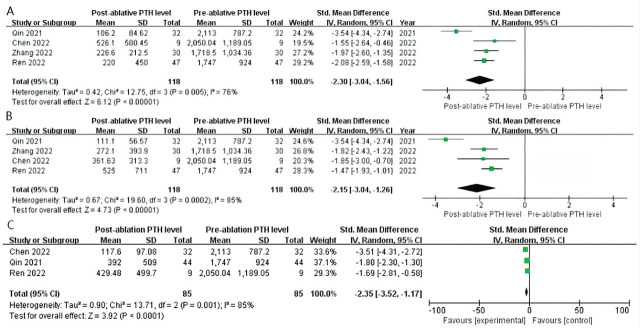
Meta-analysis and forest plot of post-RFA and pre-RFA of PTH levels. **(A)** 1 d, **(B)** 6 months, and **(C)** 12 months. PTH, parathyroid hormone; RFA, radiofrequency ablation.

All included articles reported data on PTH levels. The levels were remarkably decreased at 1 d, 6 months, and 12 months (reported by 3 of the included articles: Qin et al. [39], Ren et al. [37], and Chen et al. [36]) after RFA for SHPT: 1 d (SMD = −2.30, 95% CI = from −3.04 to −1.56, *P* < 0.0001), 6 months (SMD = −2.15, 95% CI = from −3.04 to −1.26, *P* < 0.0001), and 12 months (SMD = −2.35, 95% CI = from −3.52 to −1.17, *P* < 0.0001; **Figure 2**). The values of Cohen's *d* for 1 d, 6 months, and 12 months were 2.339, 2.194, and 2.390, respectively, and the effect size was defined as huge. The result showed significant heterogeneity (*P* < 0.0001, *I*^2^ > 76%), and the random-effects model was appropriate for this meta-analysis.

#### Calcium level

**Figure 3.**
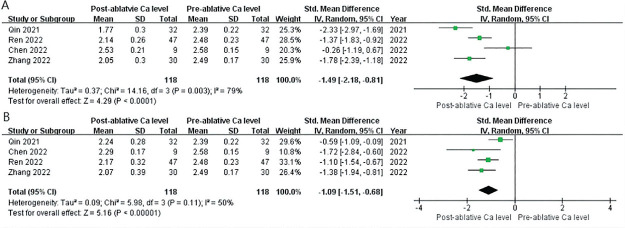
Meta-analysis and forest plot of post-RFA and pre-RFA of serum calcium levels. **(A)** 1 d and **(B)** 6 months. RFA, radiofrequency ablation.

All included articles reported serum calcium levels. The data showed that serum calcium levels were dramatically decreased at 1 d, 6 months, and 12 months after RFA: 1 d (SMD = −1.49, 95% CI = from −2.18 to −0.81, *P* < 0.0001) and 6 months (SMD = −1.09, 95% CI = from −1.51 to −0.68, *P* < 0.0001, **Figure 3**). The value of Cohen's *d* for 1 d and 6 months were 1.501 and 1.118, respectively, and the effect size was defined as very large and large. The result showed significant heterogeneity (*P* < 0.0001, *I*^2^ > 50%), and the random-effects model was appropriate for this meta-analysis.

#### Phosphorus level

**Figure 4.**
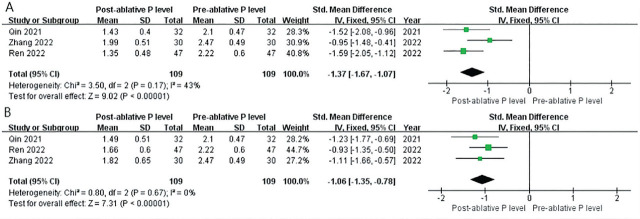
The meta-analysis, funnel plot, and forest plot of post-RFA and pre-RFA of serum phosphorus levels. **(A)** 1 d and **(B)** 6 months. RFA, radiofrequency ablation.

Serum phosphorus levels were reported by 3 of the included articles, i.e., Ren et al. [37], Zhang et al. [38], and Qin et al. [39]. The data showed that the serum phosphorus level was significantly reduced at 1 d and 6 months after RFA: 1 d (SMD = −1.37, 95% CI = from −1.67 to −1.07, *P* < 0.0001) and 6 months (SMD = −1.06, 95% CI = −1.35 to −0.78, *P* < 0.0001; **Figure 4**). The values of Cohen's *d* for 1 d and 6 months were 1.339 and 1.050, respectively, and the effect size was defined as very large and large. The fixed-effects model was used for meta-analysis owing to low heterogeneity (*P* < 0.0001, *I*^2^ < 50%).

## Complications

Different complications were reported in the included studies, and the details are shown in the following table (**Table 3**). For the side effects, in Chen et al.'s [36] report, 2 patients complained of transient hoarseness, while 5 patients complained of neck pain at 1 month and 4 patients recurrent hypercalcemia; Ren et al. [37] has the table of complications for 2 groups of patients (PTX vs. RFA) with different treatments; Zhang et al. [38]. had only the number of Recurrent laryngeal nerve (RLN) injury for 2 groups of patients (RFA vs. parathyroidectomy with autotransplantation (PTX + AT)); and Qin et al.'s [39] reported that 9 of 32 patients had hoarseness (28%) and 2 of the 9 patients coughed while drinking water, thus suggesting recurrent laryngeal nerve injuries (Post RFA). Therefore, the post complication data from PFA treatment that could be combined is as follows: hoarseness 2 of 14 posts in Chen et al. [36], 6 of 47 in Ren et al. [37], and 9 of 32 in Qin et al. [39]; hypercalcemia: 4 of 14 in Chen et al. [36] and 26 of 47 in Ren et al. [37]; and RLN injury: 5 of 30 in Zhang et al. [38] and 2 of 9 in Qin et al. [39].

**Table 3.** Combined data of side effects

	**Chen**	**Ren**	**Zhang**	**Qin**	**Total**
Hoarseness	2/14	6/47	–	9/32	17/93 (18.3%)
Hypercalcemia	4/14	26/47	–	–	30/61 (49.2%)
RLN injury	–	–	5/30	2/9	7/39 (17.9%)

## Quality assessment

### Sensitivity analyses

Sensitivity analyses of changes in the serum PTH level at 1 d, 6 months, and 12 months and serum calcium and phosphorus levels at 1 d and 6 months after RFA were performed to determine the dependability of the results. One study was excluded at a time, and the results and heterogeneity showed no difference when excluding the data from Qin et al. [39].

### Publication bias

Publication bias analyses were performed for changes in serum PTH and calcium levels at the evaluation time points of 1 d and 6 months in all included studies and for changes in serum phosphorus levels at the time points of 1 d and 6 months in Ren et al. [37], Zhang et al. [38], and Qin et al. [39]. Egger's test was used to quantify the publication bias (**Table 4**), and none of the *P*-values were < 0.05. Hence, publication bias was not statistically supported.

**Table 4.** Publication bias analyses were performed using Egger's test

**Outcomes**	**Follow-up time point**	***t***	***P***
Serum PTH	1 d	−1.313	0.320
	6 months	−0.819	0.499
	12 months	−0.849	0.552
Serum calcium	1 d	−1.942	0.192
	6 months	−0.577	0.622
Serum phosphorus	1 d	−0.652	0.632
	6 months	−0.219	0.863

PTH, parathyroid hormone.

### GRADE assessment

Each result was evaluated using GRADE assessment. Serum PTH levels were changed at 1 d, 6 months, and 12 months after RFA and were rated as moderate (+++). Serum calcium levels were varied at 1 d and 6 months after RFA and were rated as moderate (+++). Serum phosphorus levels were changed at 1 d and 6 months after RFA and were also rated as moderate (+++).

## Discussion

In this meta-analysis, post-ablation data of serum PTH, serum calcium, and serum phosphorus levels were compared with baseline values and analyzed; the results showed that all the above biochemical indicators were reduced at 1 d and 6 months and serum PTH level at 12 months after RFA.

Destruction of the parathyroid gland hyperplasia using RFA is the main reason for the decrease in PTH. Sensitivity analyses of changes in serum PTH indicated that the data from Qin et al.'s [39] study were the source of heterogeneity. On excluding these data, the heterogeneity was eliminated (*P* < 0.001, *I*^2^ = 0). The review included four articles; only Qin et al [39]. used contrast-enhanced ultrasound (CEUS) before, during, and after RFA to determine parathyroid localization and evaluate RFA performance. On the contrary, other studies used technetium-99m methoxyisobutylisonitrile, enhanced computed tomography, single-photon emission computed tomography, and high-frequency neck. In addition, Qin et al.'s [39] study showed a more significant decrease in serum PTH levels than the other studies at each follow-up time point. This result proves that imaging modalities can influence clinical outcomes, which signifies that the differences in experience among various operators in RFA procedures cause perceived heterogeneity [40]. Furthermore, this meta-analysis confirmed that RFA guided by CEUS is more feasible and useful to treat SHPT than US-guided RFA.

The reduction in serum calcium levels at 1 d and 6 months after RFA was also established in this research. Sensitivity analyses of changes in the serum calcium level indicated that the data from Qin et al.'s [39] study were the source of heterogeneity; on excluding these data, heterogeneity was eliminated at the 6-month follow-up time point evaluation (*P* < 0.001, *I*^2^ = 0). Previous research has demonstrated that thermal ablation could maintain a relatively high PTH level, decreasing the risk of hypocalcemia than PTX [41]. Moreover, compared with PTX, thermal ablation may ablate only a part of the parathyroid, which is visible on the ultrasonic device screen, but surgery results in complete excision. CEUS can precisely image the shape and extent of the parathyroid glands; the outcomes of CEUS-guided RFA are similar to those of PTX, which might explain the lower decrease in serum calcium levels in Qin et al.'s [39] research.

Complications and adverse effects were observed in all included articles. Hoarseness was the most common complication that occurred in 25 of the 118 patients (21%) after RFA but was reversed within 6 months. This could be attributed to the heat from RFA, which caused laryngeal nerve stress reaction and temporary impairment of the vocal fold mobility [42]. The liquid isolation zone was established in all studies to prevent recurrent laryngeal nerve injury, and research has shown that thermal ablation has a lower incidence rate of hoarseness than PTX [41]. However, owing to the anatomical proximity of the recurrent laryngeal nerve to the parathyroid gland, it can easily be injured during surgery in the adjacent site. Moreover, the complexities of neck anatomy and parathyroid glands make it challenging to avoid hoarseness. Other complications reported in the 4 articles included hypocalcemia (27%), severe hypocalcemia (10%), recurrence (6%), neck pain (4%), artery injury, and hematoma (1%). Zhang et al. [38] described severe hypocalcemia and recurrence following RFA, but the incidence rates of severe hypocalcemia did not differ significantly between RFA and PTX. Another research [41] concluded that the rate of persistence or recurrence in thermal ablation was higher than that in PTX. Nonetheless, the articles included in this meta-analysis did not show such a trend.

Referring to the Kidney Disease Outcome Quality Initiative (K/DOQI) guidelines [43], Kidney Disease Improving Global Outcomes (KDIGO) guidelines [44], and the guidelines or expert consensus formulated by the Chinese Society of Nephrology, surgery is preferred for refractory Hyperparathyroidism (HPT). However, patients suffering from CKD may have a high rate of intolerance to general anesthesia or cannot accept conventional surgical treatment. Clinicians prefer to use ultrasound-guided thermal ablation to treat SHPT because it is a less invasive therapeutic intervention and can provide a shorter recovery period with efficacy. Thermal ablation has recently been used as an alternative to SHPT in older patients, especially in those with poor cardiopulmonary function and low tolerance to conventional surgery. Based on the available research, this study is the first meta-analysis to evaluate the effectiveness and safety of US-guided RFA for SHPT.

Limitations cannot be avoided in this meta-analysis. As the median follow-up time was 6 months and the longest follow-up time was 24 months, a longer follow-up period is needed to confirm the effect of RFA. Another limitation is the exclusion of RCTs due to unrelated interventions and incomplete data and the inclusion of only retrospective studies in this meta-analysis; hence, there could have been a certain bias in patient selection. Moreover, as all included studies were from China, caution is required in generalizing the conclusions to other ethnicities.

Although nearly half of all new cancer cases are in Asia [10] and the population prevalence of maintenance dialysis treatment in China is estimated to be 79 per million population [45], it is still difficult to collect suitable cases for research. One of the reasons is that the 5-year mortality rate for CKD stage 4 is 58.8% [46]. Moreover, lower socioeconomic status is possibly responsible for a higher rate of PTX [47]. Despite the cost of RFA being lower than that of PTX [37], the application of RFA is not popularized in clinical practice, and the learning curve of RFA remains a limitation for clinical penetration.

## Conclusions

US-guided RFA is an effective and safe treatment for hyperparathyroidism secondary to CKD. RFA can be used as a new strategy to treat SHPT due to the decrease in serum PTH, calcium, and phosphorus levels after the procedure. Hoarseness is the most common complication but is usually reversed within 6 months after the ablation.
